# Thiotemplated Polyketide Chain Fusion and Reductive Cyclization Build the Reactive Butenolide Core of Malleicyprol

**DOI:** 10.1002/anie.202504485

**Published:** 2025-06-09

**Authors:** Jonas Fiedler, Leo Dumjahn, Mie Ishida‐Ito, Felix Trottmann, Keishi Ishida, Christian Hertweck

**Affiliations:** ^1^ Department of Biomolecular Chemistry Leibniz Institute for Natural Product Research and Infection Biology (HKI) Beutenbergstraße 11a, 07745 Jena Germany; ^2^ Institute of Microbiology Faculty of Biological Sciences Friedrich Schiller University Jena, 07743 Jena Germany

**Keywords:** Biosynthesis, Enzymes, Natural products, Virulence factor

## Abstract

Malleicyprol, a virulence factor of notorious animal and human pathogens of the *Burkholderia pseudomallei* (BP) group, features a molecular cyclopropanol warhead linked to a reactive butenolide core. Biosynthetic considerations suggested that this heterocycle was formed by the merger of two individual polyketide chains, but the precise mechanism has remained elusive. By combining chemical synthesis, complete in vitro reconstitution of the biotransformation, and mutational analysis, we show that two individually generated polyketide chains are joined by a noncanonical condensation domain of the PKS–NRPS hybrid synthetase BurF, which forms an ester bond. By mutagenesis, biochemical assays, and trapping of the aldehyde generated from a substrate surrogate, we found that the terminal reductase domain mediates a reductive chain release with concomitant ring formation. The feasibility of the proposed Knoevenagel‐type intramolecular cyclization into the butenolide moiety was confirmed by a biomimetic synthesis of malleicyprol. The elucidation of the unprecedented thiotemplated butenolide biosynthesis by head‐to‐head fusion of two polyketide chains not only expands the synthetic biology toolbox but may also inspire the development of antivirulence strategies against BP pathogen infections.

## Graphical abstract

Malleicyprol, a virulence factor of notorious animal and human pathogens of the *Burkholderia pseudomallei* (BP) group, results from the fusion of two individual polyketide chains. The noncanonical condensation domain of the PKS–NRPS hybrid synthetase BurF forms an ester bond, and the terminal reductase domain mediates a reductive chain release followed by ring formation. Biomimetic synthesis of malleicyprol confirms the feasibility of the proposed Knoevenagel‐type intramolecular cyclization process that forms the butenolide ring.

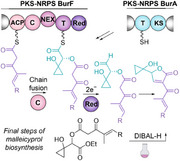

## Introduction

A large number of microbial polyketides, which play important roles in ecology, agriculture, and medicine, are assembled by molecular assembly lines.^[^ ^1^ ^]^ In a highly programmed manner, multimodular polyketide synthases (PKS) generate diverse polyketide chains from simple acyl and malonyl building blocks;^[^ ^1^ ^]^ as all intermediates are bound as thioesters to carrier proteins, the enzyme systems are referred to as thiotemplates.^[^ ^2^, ^3^ ^]^ Structural diversity results from the choice of alternative starter and extender units,^[^ ^4^ ^]^ the degree of reduction of the β‐carbonyls,^[^ ^1^ ^]^ the chain lengths, and the diverse off‐loading mechanisms.^[^ ^5^ ^]^ Almost exclusively, the unidirectional propagation of the growing carbon backbone leads uniformly to linear chains, which are transformed into various shapes by downstream processes such as (poly)cyclizations.^[^ ^6^ ^]^ Beyond such tailoring reactions, various assembly lines have evolved enzymatic mechanisms to branch the polyketide chain by Michael additions^[^ ^7^ ^]^ or isoprenoid‐like modifications.^[^ ^8^ ^]^ Alternatively, unusual substitutions can be introduced during chain termination, condensing a β‐keto thioester with glycerate (by FabH‐like enzymes)^[^ ^9^ ^]^ or dihydroxyacetone (by AfsA‐like enzymes)^[^ ^10^, ^11^ ^]^ to produce tetronates and butyrolactones/butenolides, respectively. Notable examples include the tetronates RK‐682^[^ ^9^ ^]^ and tetromadurin,^[^ ^12^ ^]^ the butenolides gladiofungin^[^ ^10^ ^]^ and styrolide A,^[^ ^13^ ^]^ and the butyrolactone A‐factor.^[^ ^14^ ^]^ There are only very few known polyketide synthases that create unique molecular scaffolds by the head‐to‐head fusion of two individual polyketide chains. Important examples are the bacterial biosynthetic pathways to corallopyronin and myxopyronin, in which ketoacyl synthase III homologs (CorB, MxnB) generate the pharmacophoric pyrone moiety.^[^ ^15^, ^16^ ^]^ The furanone moiety of the fungal metabolite gregatin is generated by a Claisen‐type polyketide chain fusion catalyzed by an α/β hydrolase, GrgF.^[^ ^17^ ^]^

**Figure 1:**
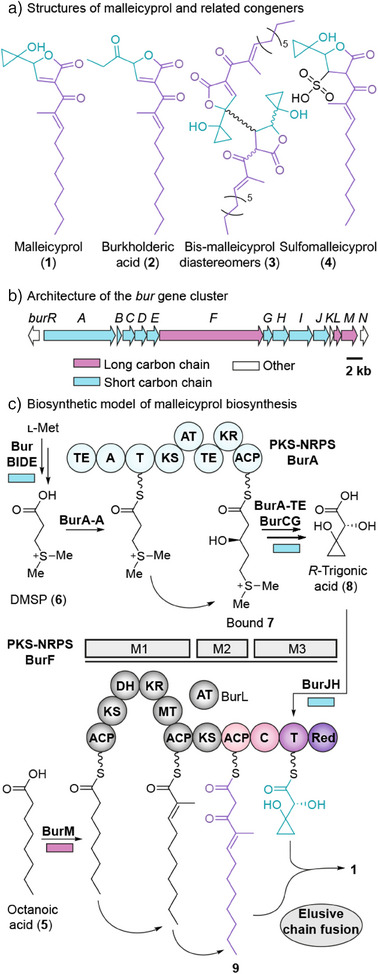
Structures and biosynthesis of the malleicyprol virulence factor complex. a) Structures of malleicyprols and related congeners. The molecules are colored to represent that they originate from two chains, a short carbon chain (light blue) and a long carbon chain (purple). b) Architecture of the malleicyprol biosynthesis gene cluster. c) Model of malleicyprol biosynthesis, outlining the merger of two individually formed polyketide chains. A more detailed scheme is shown in Figure S1. M, module; ACP, acyl carrier protein; KS, ketosynthase; DH, dehydratase; KR, ketoreductase; MT, methyltransferase; AT, acyltransferase; C, condensation domain; T, thiolation domain; Red, reductase.

A different type of polyketide chain fusion has been implicated in the biosynthesis of malleicyprol (**1**),^[^ ^18^, ^19^ ^]^ an important virulence factor of human‐ and animal‐pathogenic bacteria that belong to the *Burkholderia pseudomallei* group.^[^ ^20^ ^]^ A key structural feature of **1** is the butenolide core with a cyclopropanol substitution. The strained ring functions as a molecular warhead that likely undergoes a radical ring opening to form the inactive congener burkholderic acid (*syn*. malleilactone) (**2**).^[^ ^18^, ^19^, ^20^, ^21^ ^]^ In addition, the butenolide ring itself is highly reactive; it dimerizes to bis‐malleicyprol (**3**) due to its ambident donor/acceptor properties and readily forms the sulfite adduct sulfomalleicyprol (**4**) (Figure 1a).^[^ ^19^ ^]^

Previous biosynthetic studies have suggested that **1** is assembled from two carbon chains, a short one and a long one, that are individually produced by two different polyketide synthases, BurA^[^ ^22^ ^]^ and BurF, encoded in the *bur* gene locus (Figure 1b). BurF is a multimodular thiotemplate that first generates the long carbon chain by processing medium‐sized fatty acids (mainly octanoic acid, **5**, Figure 1c) and catalyzes two extensions with malonyl‐CoA.^[^ ^23^ ^]^ The monomodular BurA, on the other hand, extends the unusual l‐methionine‐derived starter unit dimethylsulfoniumpropionate (DMSP) (**6**) to produce *S*‐gonyol (**7**),^[^ ^22^ ^]^ which is converted to *R*‐trigonic acid (**8**) by means of a hydroxylase (BurC) and a repurposed ketol‐acid reductoisomerase (BurG) (Figures 1c and S1 for a more detailed scheme).^[^ ^24^ ^]^ Subsequently, a two‐component system (BurJ and BurH) loads α‐hydroxy acid **8** onto the thiolation (T) domain of a noncanonical module of BurF that resembles a nonribosomal peptide synthetase.^[^ ^25^ ^]^ However, the precise manner in which the two chains are fused and how the butenolide is constructed remains to be clarified.

Here we elucidate the final biosynthetic steps in mallei‐cyprol biosynthesis, characterize an unusual butenolide‐forming thiotemplate system, and dissect the reaction sequence that not only inspired a biomimetic synthesis of malleicyprol but also provides the basis for pathway engineering of butenolide systems.

## Results and Discussion

**Figure 2:**
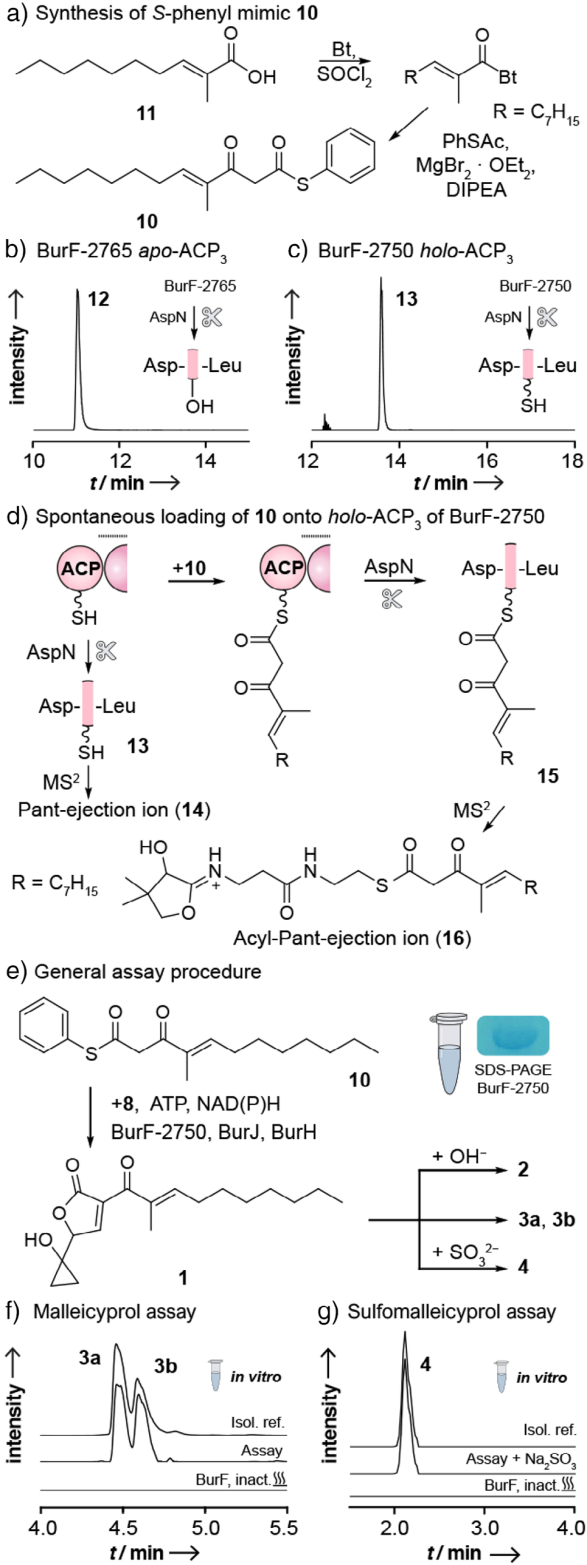
In vitro reconstitution of malleicyprol biosynthesis. a) Synthetic route to S‐phenyl β‐keto thioester **10**. b) Proteolytic digestion of BurF‐2765 using AspN shows that its ACP_3_ is not phosphopantetheinylated (extracted ion chromatogram (EIC): *m/z* 471.0142; [M + 4H]^4+^). c) Proteolytic digestion of BurF‐2750 using AspN shows that its ACP_3_ is converted to the *holo* form (EIC: *m/z* 556.0356; [M + 4H]^4+^). d) Incubation of BurF‐2750 with **10** before digestion leads to spontaneous loading of **10** onto the protein. e) BurF‐2750 forms **1** from **10** and **8** using NADPH as a cofactor. Isomerization and dimerization of **1** leads to bis‐malleicyprol (observed as diastereomers **3a** and **3b**). Basic treatment of the enzymatic reaction with NaOH leads to the formation of burkholderic acid (**2**), and addition of sodium sulfite (Na_2_SO_3_) yields sulfomalleicyprol (**4**). See Supporting Information for full SDS‐PAGE. f) HPLC‐HRMS analysis of bis‐malleicyprol (EIC: *m/z* 611.3589; [M − H]^−^) formation using standard assay conditions. g) HPLC‐HRMS analysis of sulfomalleicyprol (EIC: *m/z* 387.1483; [M − H]^−^) formation after addition of Na_2_SO_3_. Bt, benzotriazole; inact., inactivated.

The malleicyprol assembly line, bioinformatically deduced from the *bur* gene cluster, suggests that the terminal module, consisting of condensation (C), thiolation (T), and reductase (Red) domains, would merge and cyclize two individually generated polyketide chains. To test this, we aimed to reconstitute these biosynthetic steps in vitro. Therefore, we heterologously produced a truncated variant of BurF (BurF‐2765, based on BurF amino acid numbering) containing the terminal module and the upstream ACP_3_.^[^ ^25^ ^]^ As substrate, we synthesized a mimic of the proposed biosynthetic intermediate **9** that would be tethered to the phosphopantetheinyl‐ACP_3_ (Figure 1c). Specifically, we prepared a *S*‐phenyl β‐keto thioester analogue **10** that was assumed to undergo an in vitro transacylation reaction. In brief, the branched α,β‐unsaturated acid **11** was generated by Wittig olefination of octanal, followed by deprotection.^[^ ^26^ ^]^ The acid **11** was then activated by benzotriazole (Bt) and subjected to a crossed Claisen condensation^[^ ^27^ ^]^ with *S*‐phenyl thioacetate (PhSAc), yielding the desired pathway surrogate **10** (Figure 2a, for details see Figure S2, overall yield: 42 %). The second building block, *rac*‐trigonic acid (**8**), was synthesized according to an established protocol^[^ ^24^ ^]^ and was enzymatically loaded onto the BurF T domain by BurJ and BurH.^[^ ^25^ ^]^

Initial attempts to reconstitute the biotransformation in vitro were hampered by incomplete substrate loading. High‐performance liquid chromatography high‐resolution mass spectrometry (HPLC‐HRMS) measurements of the proteo‐lytic products of AspN‐digested BurF‐2765 confirmed the successful loading of **8** onto the T domain, as observed previously.^[^ ^25^ ^]^ However, we found that peptide **12** (*m*/*z* 471.0142; [M + 4H]^4+^) (Figure 2b), derived from ACP_3_, contained the serine residue for PPant attachment (Figure S3A) but lacked the PPant modification (Figure S3B,C). By protein fold prediction using the Phyre^2^ server,^[^ ^28^ ^]^ we realized that the truncation of BurF‐2765 apparently shortened the ACP_3_ by several amino acids (Figure S4). This incomplete folding could result in an impaired recognition by the phosphopantetheinyl transferase (PPTase).

To test this possibility, we designed a variant, BurF‐2750, with fifteen additional amino acids at the N‐terminus that are predicted to form helix 1 of ACP_3_ (Figure S4). We heterologously produced His‐tagged BurF‐2750 in *Escherichia coli*, coexpressing the PPTase gene *svp* ^[^ ^29^ ^]^ to generate the *holo* form in vivo, and purified the product by affinity chromatography. By HPLC‐HRMS analysis of AspN‐digested BurF‐2750, we detected peptide **13** (*m*/*z* 556.0356; [M + 4H]^4+^) (Figure 2c), and MS^2^ revealed its diagnostic Pant‐ejection ion^[^ ^30^ ^]^ **14** (*m*/*z* 261.1268; [M + H]^+^) (Figure S5), demonstrating that the new BurF variant contains ACP_3_ in the *holo*‐form (Figures 2d and S5). When we incubated BurF‐2750 with mimic **10**, AspN digestion led to peptide **15** (*m*/*z* 716.9591; [M + 5H]^5+^) with the expected mass shift, indicating that **10** was bound to the thiotemplate. We verified the identity of **15** by MS^2^ analysis, which showed the corresponding acyl‐Pant ejection ion (**16**, Figures 2d and S5).

These experiments taught important lessons. First, it is a reminder of how important it is to maintain the integrity of structural recognition sites in fragments of thiotemplate assembly lines in order to warrant their transformation into the *holo* form. Second, thioester mimic **10** cannot bypass the PKS module; the polyketide intermediate must first be covalently bound to ACP_3_ to serve as a substrate of the downstream C domain. Finally, the polyketide chain of phenyl thioester **10** can be attached to the PPant chain of ACP_3_ (Figure 2d) by spontaneous transacylation.^[^ ^31^ ^]^

For the in vitro reconstitution of the polyketide chain merger, we first loaded **8** onto the T domain of BurF‐2750 with BurJ, BurH, and ATP. Then, we added the phenyl thioester surrogate **10** for ACP_3_ self‐loading and NADH or NADPH as cofactor of the Red domain, mediating the expected reductive release (Figure 2e). HPLC‐HRMS monitoring of the enzyme assay showed the formation of a product with *m/z* 611.3589 [M − H]^−^, which was identified to be **3** by comparison to an authentic reference compound (Figures 2f and S6).^[^ ^19^ ^]^ Compound **3** is observed as mixture consisting of two main diastereomers (**3a** and **3b**).^[^ ^19^ ^]^ Furthermore, we observed the formation of **1** (*m/z* 305.1758; [M − H]^−^) (Figure S7) that coexists in a chemical equilibrium^[^ ^19^ ^]^ with **3**. Basic treatment of the assay mixture generated **2** (*m/z* 305.1758; [M − H]^−^) (Figure S8),^[^ ^19^ ^]^ and addition of sodium sulfite to the assay led to the formation of the sulfonated adduct **4** (*m/z* 387.1483; [M − H]^−^) (Figures 2g and S9).^[^ ^19^ ^]^ The successful biotransformation indicated that the terminal BurF module is sufficient to promote the final steps of malleicyprol biosynthesis in vitro.

**Figure 3:**
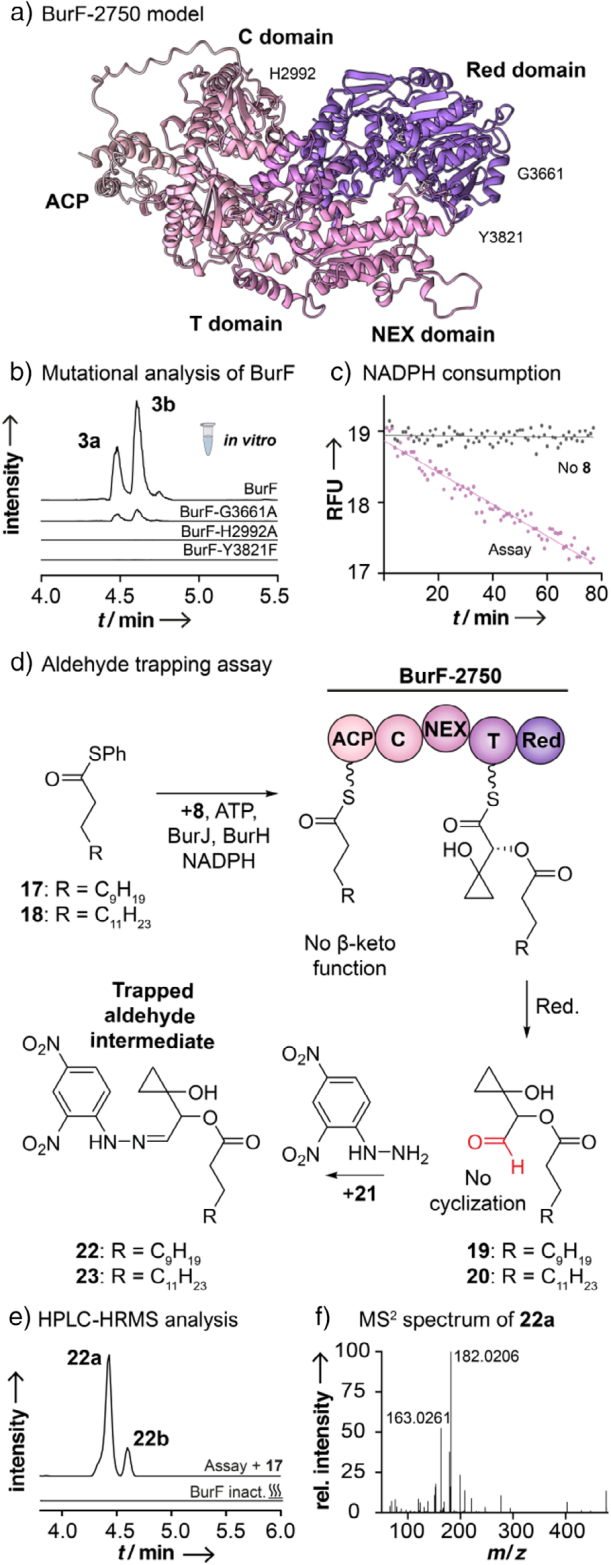
Biochemical analysis of BurF. a) Structural model of BurF showing the multidomain architecture. (See Supporting Information for more detailed snapshots and superimpositions with characterized proteins.) b) Site‐directed mutagenesis of key residues of BurF abolishes or strongly reduces product formation (EIC: *m/z* 611.3589; [M − H]^−^). c) NADPH is rapidly oxidized during the catalysis of BurF (*λ*_exc_  =  340 nm and *λ*_emm_  =  460 nm). d) Aldehyde trapping using the derivatization reagent DNPH (**21)**. e) HPLC‐HRMS analysis of formation of **22** (EIC: *m/z* 477.2355; [M − H]^−^). f) MS^2^ spectrum of **22a** shows typical fragments of DNPH derivatives. RFU, relative fluorescence units.

To gain a more in‐depth insight into the multistep enzymatic conversion to the butenolide ring, we first created a structural model of the BurF fragment using AlphaFold 3 (Figure 3a).^[^ ^32^ ^]^ The model shows the expected T, C, and Red domains. The C domain forms a head‐to‐tail pseudodimer with an N‐lobe containing the typical active site motif (HH_2992_xxxDx) for peptide bond formation (Figure S10).^[^ ^33^ ^]^ The model of the Red domain shows the typical fold of short‐chain dehydrogenases/reductases (SDR‐type enzymes)^[^ ^34^ ^]^ and the characteristic helix–turn–helix motif of terminating Red domains (Figure S11).^[^ ^35^ ^]^ Unexpectedly, AlphaFold 3 also predicted a previously overlooked domain of unknown function (DUF, 244 amino acids, Figure S12) located between the C and T domains.

To functionally characterize the individual domains, we first targeted the C domain by introducing a mutation in the active site (His2992Ala). This mutation completely abolished product formation (Figure 3b), indicating that the BurF C domain catalyzes the chain‐merging ester bond formation in analogy to peptide formation in canonical C domains of NRPS (Figure S13).^[^ ^36^ ^]^ A sequence analysis of the BurF C domain using the NaPDoS2 server^[^ ^37^ ^]^ shows that it groups with ^L^C_L_‐type C domains (Figure S14), like most other in vitro characterized (SgcC5, VioA, CesAB, VlmAB, CrpD, and FkbP) or predicted (MdpC5, KedY5, RapP) ester‐forming C domains (Table S1). This suggests that these C domains retain key catalytic residues characteristic of ^L^C_L_‐type enzymes. Interestingly, BurF and most of the other mentioned C domains are assigned to the ester‐forming SgcC5‐type, using a more comprehensive classification based on core motif identification (Table S2).^[^ ^38^ ^]^ This highlights the importance of considering these motifs when analyzing and engineering specialized C domains.

Analysis of the DUF initially did not reveal any conserved motifs typically associated with PKS or NRPS assembly lines. However, with the help of Foldseek,^[^ ^39^ ^]^ we noted similarities of the DUF with members of the FkbH‐like protein family (Table S3).^[^ ^40^ ^]^ More specifically, this similarity corresponds to the previously described N‐terminal extension (NEX) of the phosphatase subdomain within some of these proteins (Figure S15).^[^ ^41^ ^]^ Consequently, we have designated the DUF as “NEX” domain.

As NEX domains may play a role in the transfer of bound glycerate units onto carrier proteins,^[^ ^41^ ^]^ we assume that this function could extend to BurF by mediating an interaction to the transfer protein BurH^[^ ^25^ ^]^ in the translocation of **8** onto the BurF‐T domain. As functional analyses were hampered by the lack of identifiable functional residues suitable for mutational analysis, we inferred the functional relevance of the BurF NEX domain by generating a sequence similarity network^[^ ^42^ ^]^ (Figure S16). Interestingly, we found orthologous NEX domains exclusively in multidomain proteins associated with specialized metabolism, where they are commonly coupled with a phosphatase subdomain and an acyltransferase subdomain of FkbH‐like proteins (95 % of orthologs, see Figure S16). It is conceivable that BurF has lost such subdomains during assembly line evolution.^[^ ^43^, ^44^ ^]^

Analysis of the terminal Red domain revealed a C‐terminal domain predicted for substrate recognition, an N‐terminal subdomain with a Rossmann fold containing the fingerprint motif (G_3661_xxGxxG) for NAD(P)H binding,^[^ ^45^ ^]^ and the conserved catalytic residues (Y_3821_xxxK) for thioester reduction.^[^ ^46^ ^]^ To test for cofactor dependence, we performed enzyme assays using either NADH or NADPH as reducing agents. We found that NADPH is the preferred cofactor (Figure S17), which is in agreement with the NADPH‐specific βαβ‐motif of the Red domain predicted by the Rossmann toolbox server (Table S4).^[^ ^47^ ^]^ In enzyme assays with BurF‐2750 and the polyketide substrates, we monitored the rapid decrease of the fluorescence signal that corresponds with NADPH consumption (Figure 3c) and production of the malleicyprols. Mutating the NADPH binding pocket (Gly3661Ala) in BurF‐2750 resulted in drastically reduced activity (Figure 3b). Nonetheless, small amounts of **3** were still detectable by HPLC‐HRMS (11 ± 4 % of relative peak area, Figure S18), indicating that the mutant can still consume NADPH, yet far less efficiently.

Typically, Red domains reduce bound thioesters to aldehydes (2e^−^ reduction, as in the biosynthesis of 3‐methylorcinaldehyde^[^ ^48^ ^]^ or linear gramicidin^[^ ^49^ ^]^) or alcohols (4e^−^ reduction, as in the biosynthesis of glycopeptidolipids^[^ ^35^ ^]^ or myxalamid^[^ ^46^ ^]^) by employing a catalytic triad containing a tyrosine residue.^[^ ^50^ ^]^ We created a variant of BurF‐2750 with a mutation in the catalytic site (Tyr3821Phe) and tested the impact in an in vitro assay. Notably, the mutated enzyme does not produce any malleicyprols (Figure 3b), validating the reaction mechanism described for this enzyme family (Figure S19) and confirming that the reduction is a critical step in malleicyprol formation.

The immediate product of the *bur* assembly line would be an aldehyde that could undergo an intramolecular Knoevenagel‐type cyclization. To provide more experimental support for this idea, we sought to trap the aldehyde intermediate. Therefore, we synthesized substrate mimics (**17**, derived from lauric acid and **18**, derived from myristic acid) lacking the β‐keto group and individually subjected the analogues to the enzyme assay with BurF‐2750 (Figure 3d). We captured aldehydes **19** and **20** by adding the derivatization reagent 2,4‐dinitrophenylhydrazine (DNPH) (**21**) and detected the corresponding dinitrophenylhydrazones **22** and **23** by HPLC‐HRMS (observed as *E*‐*Z* isomers,^[^ ^51^ ^]^ Figures 3e and S20). MS^2^ analyses showed that both conjugates match the expected *m/z* and release fragments that are characteristic for the DNPH moiety (Figures 3f and S20),^[^ ^52^ ^]^ thus, pointing to the formation of aldehyde intermediates.

**Figure 4:**
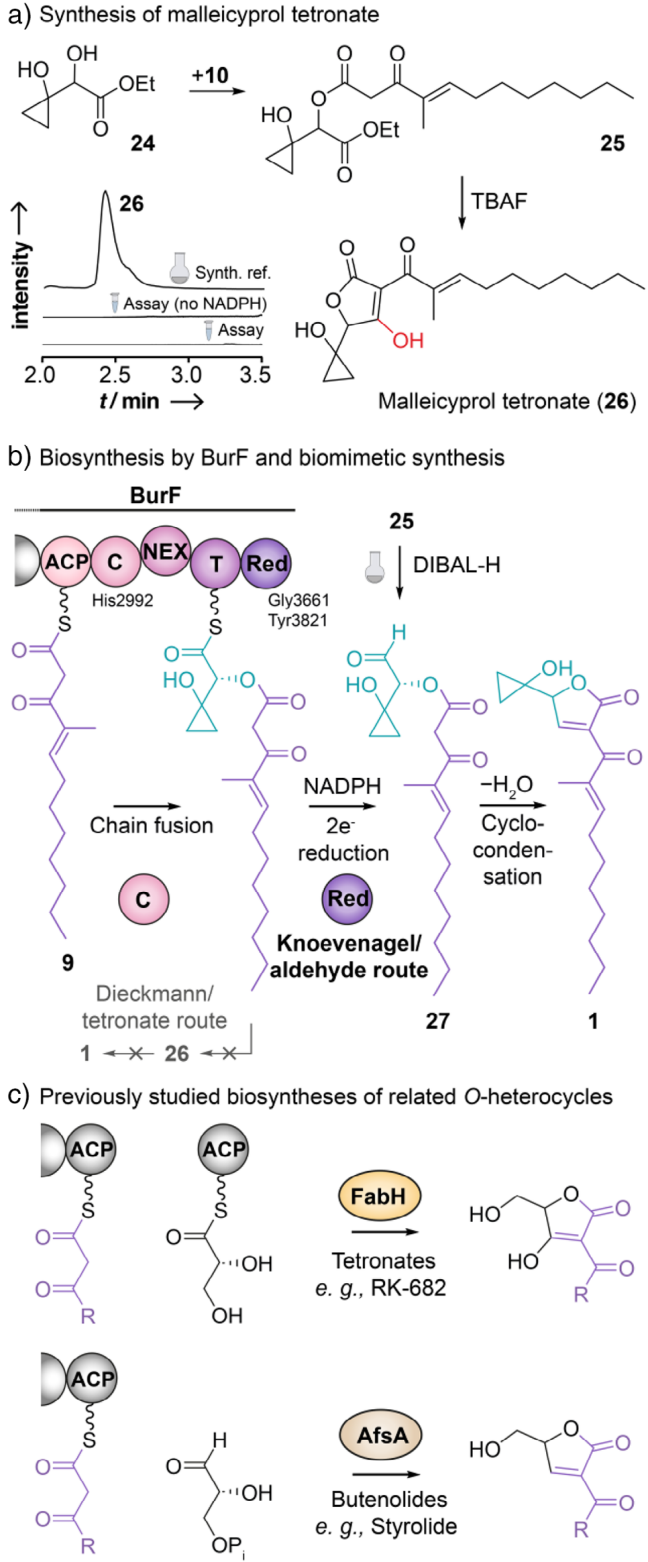
Final steps of malleicyprol biosynthesis. a) Malleicyprol tetronate (**26**) synthesis and analysis of BurF assay for this compound (EIC: *m/z* 321.1707; [M − H]^−^) shows that **26** is no intermediate during the formation of malleicyprol. b) C domain of BurF catalyzes ester bond formation between enzyme‐bound **8** and bound intermediate **9**. Subsequently, the reductase domain reduces the bound thioester to an aldehyde that cyclizes to the butenolide **1**. Addition of the hydride donor DIBAL‐H to **25** generates **1** in a biomimetic fashion. c) Characterized assembly strategies for tetronates and butenolides in specialized metabolism.

Nonetheless, one has to consider that the timing of the reduction could differ when the native substrate is transformed. Indeed, various fungal reductase‐like domains (R*) are nonredox enzymes that catalyze Dieckmann‐type reactions, yielding tetramates as products (e.g., biosynthesis of equisetin,^[^ ^53^ ^]^ cyclopiazonic acid,^[^ ^54^ ^]^ or tenellin^[^ ^55^ ^]^). To investigate whether an analogous tetronate could serve as an intermediate *en route* to **1** (Figure S21), we prepared a synthetic tetronate reference. To do so, we coupled *S*‐phenyl thioester **10** with the ethyl‐protected trigonyl building block **24** using silver trifluoroacetate to generate oxoester **25**. The subsequent TBAF‐mediated cyclization led to the mallei‐cyprol tetronate (**26**) (Figure 4a). However, HPLC‐HRMS monitoring of the enzyme assay did not show any signal matching the synthetic reference (*m/z* 321.1707; [M − H]^−^). Even in assays performed in the absence of NADPH, which would favor the accumulation of **26**, the Dieckmann product could not be observed. Instead, we detected traces of a compound (*m/z* 339.1813; [M − H]^−^, Figure S22) that matches the expected *m*/*z* of the carboxylic acid, which would result from spontaneous thioester hydrolysis. Notably, carboxylic acids are typical shunt products resulting from premature release of polyketide intermediates from *trans*‐AT thiotemplate systems in which downstream processes are blocked.^[^ ^56^, ^57^, ^58^, ^59^ ^]^

Although our findings indicate the improbability of a potential Dieckmann‐type reaction, the feasibility of a spontaneous reductive cyclization remained to be demonstrated. We therefore devised a biomimetic synthesis of malleicyprol, in which the butenolide ring is formed by such an approach. Specifically, we treated ethyl ester **25** (see above) with DIBAL‐H to produce the corresponding aldehyde **27**. HPLC‐HRMS analysis showed that **1** (*m/z* 305.1758; [M − H]^−^) is formed immediately after the addition of the hydride donor (Figures 4b and S23). As was the case in our in vitro studies, the transient aldehyde **27** was not observed, indicating the propensity of the intermediate to cyclize immediately to **1**. Concentrating the reaction mixture yielded **3**, again highlighting the reactive nature of the butenolide core and the chemical equilibrium between the two compounds.

## Conclusion

This study sheds light on the final steps in the biosynthesis of malleicyprol, a crucial virulence factor produced by notorious pathogens of the *Burkholderia pseudomallei* group, which cause severe diseases in humans and animals. We elucidate the intricate fusion of two individual polyketide chains that gives rise to the toxin's reactive butenolide core (Figure 4b).

The unusual PKS–NRPS hybrid synthetase BurF merges an elongated fatty acid–polyketide intermediate and a bound α‐hydroxy acid by means of a noncanonical C domain. As such, this C domain expands the small family of characterized ester‐forming C domains. Opposed to the characterized free‐standing SgcC5‐type homolog,^[^ ^60^ ^]^ the BurF C domain is an integrated part of a multimodular thiotemplate system as in depsipeptide synthetases. Although its general architecture and reactivity is reminiscent of typical C domains, the BurF C domain is unique in accepting a fatty acid–polyketide hybrid as substrate.

Several lines of evidence, including in vitro assays, mutagenesis, and intermediate trapping, demonstrate that the terminal Red domain promotes a reductive release of the thioester, yielding a transient aldehyde intermediate that undergoes an intramolecular Knoevenagel‐type cyclization. Chemical synthesis and assay monitoring shows that a Dieckmann‐type release mechanism via a tetronate can be excluded. As such, the thiotemplated chain merger and reductive cyclization represent a novel route to butenolides that markedly differs from previously studied avenues to *O*‐heterocycles (Figure 4c).^[^ ^9^, ^10^, ^11^, ^12^, ^13^ ^]^ Interestingly, the butenolide pathway of the *bur* assembly line is functionally related to pyrrolinone formation in fungal cytochalasin biosynthesis.^[^ ^61^, ^62^ ^]^ Our biomimetic emulation of reductive release and subsequent cyclocondensation not only confirmed the model of malleicyprol biosynthesis but also marks the first synthesis of **1**.

Our in vitro pathway reconstitution involves three enzymes, a total of seven reactions, and completes the investigation of the biosynthetic pathway of malleicyprol from its initial building blocks l‐methionine and octanoic acid. The work offers detailed insights into the functional interplay between five PKS–NRPS domains, representing a significant advancement in understanding thiotemplated assembly lines. Furthermore, the reconstitution of these key steps in malleicyprol biosynthesis identified a novel assembly line module that can be employed for synthetic biology approaches to introduce reactive moieties into polyketides. Finally, detailed insight into the biosynthesis of the notorious pathogenicity factor may pave the way for designing specific inhibitors to serve as antivirulence therapeutics.

## Author Contributions

J.F., F.T., K.I., and C.H. designed research. J.F., L.D., and M.I.‐I. performed research. J.F., L.D., and M.I.‐I. analyzed data. J.F. and C.H. wrote the manuscript.

## Conflict of Interests

The authors declare no conflict of interest.

## Supporting information



Supporting Information

## Data Availability

The data that support the findings of this study are available in Supporting Information of this article.
